# Pulsed Dose Rate Brachytherapy of Lip Carcinoma: Clinical Outcome and Quality of Life Analysis

**DOI:** 10.3390/cancers13061387

**Published:** 2021-03-19

**Authors:** Radouane El Ayachy, Roger Sun, Kanta Ka, Adrien Laville, Anne-Sophie Duhamel, Anne Tailleur, Isabelle Dumas, Sophie Bockel, Sophie Espenel, Pierre Blanchard, Yungan Tao, Stéphane Temam, Antoine Moya-Plana, Christine Haie-Meder, Cyrus Chargari

**Affiliations:** 1 Radiation Oncology Department, Gustave Roussy Cancer Campus, Université Paris-Saclay, 114 rue Edouard Vaillant, 94800 Villejuif, France; radouane.el-ayachi@gustaveroussy.fr (R.E.A.); roger.sun@gustaveroussy.fr (R.S.); kanta.ka@gustaveroussy.fr (K.K.); adrien.laville@gustaveroussy.fr (A.L.); annesophie.duhamel-oberlander@gustaveroussy.fr (A.-S.D.); anne.tailleur@gustaveroussy.fr (A.T.); isabelle.dumas@gustaveroussy.fr (I.D.); sophie.bockel@gustaveroussy.fr (S.B.); sophie.espenel@gustaveroussy.fr (S.E.); Pierre.blanchard@gustaveroussy.fr (P.B.); yungan.tao@gustaveroussy.fr (Y.T.); christine.haiemeder@gustaveroussy.fr (C.H.-M.); 2 Head and Neck Surgery Department, Gustave Roussy, 94800 Villejuif, France; stephane.temam@gustaveroussy.fr (S.T.); antoine.moya-plana@gustaveroussy.fr (A.M.-P.); 3 Centre de Cancérologie, Département d’Oncologie Radiothérapie, Charlebourg la Défense, 92250 La Garenne Colombes, France; 4 INSERM1030 Radiothérapie Moléculaire et Innovations Thérapeutiques, Université Paris-Saclay, 94800 Villejuif, France

**Keywords:** brachytherapy, lip cancer, pulsed dose rate, quality of life, radiation therapy, radiotherapy

## Abstract

**Simple Summary:**

Lip cancer accounts for 25–30% of all oral cancers, with 23,000 new cases per year in the world. Carcinomas of the lip can be successfully treated with different methods: surgery, external beam radiotherapy (EBRT) and brachytherapy. The choice of the treatment depends on the tumor size, location and expected functional and esthetic results with each option, but also depends on treatment type accessibility. There are no randomized studies comparing these different treatment strategies. In this article, we investigated the complications and outcomes of patients treated with interstitial pulsed dose rate brachytherapy in our institution.

**Abstract:**

Purpose: Lip carcinoma represents one of the most common types of head and neck cancer. Brachytherapy is a highly effective therapeutic option for all stages of lip cancers. We report our experience of pulsed dose rate brachytherapy (PDR) as treatment of lip carcinoma. Methods and Materials: this retrospective single center study included all consecutive patients treated for a lip PDR brachytherapy in our institution from 2010 to 2019. The toxicities and outcomes of the patients were reported, and a retrospective quality of life assessment was conducted by phone interviews (FACT H&N). Results: From October 2010 to December 2019, 38 patients were treated in our institution for a lip carcinoma by PDR brachytherapy. The median age was 73, and the majority of patients presented T1-T2 tumors (79%). The median total dose was 70.14 Gy (range: 60–85 Gy). With a mean follow-up of 35.4 months, two patients (5.6%) presented local failure, and seven patients (19%) had lymph node progression. The Kaplan–Meier estimated probability of local failure was 7.2% (95% CI: 0.84–1) at two and four years. All patients encountered radiomucitis grade II or higher. The rate of late toxicities was low: three patients (8.3%) had grade II fibrosis, and one patient had grade II chronic pain. All patients would highly recommend the treatment. The median FACT H&N total score was 127 out of 148, and the median FACT H&N Trial Outcome Index was 84. Conclusions: This study confirms that an excellent local control rate is achieved with PDR brachytherapy as treatment of lip carcinoma, with very limited late side effects and satisfactory functional outcomes. A multimodal approach should help to improve regional control.

## 1. Introduction

Lip cancer accounts for 25–30% of all oral cancers, with 23,000 new cases per year in the world, an age-standardized incidence rate of 0.3 per 100,000 [1]. The majority of lip cancers (19.2% of all incident cases) occurs in Central and Eastern Europe with a male:female (M:F) rate ratio of about 2.5. Most cases occur in people aged 60 or over [2]. Tobacco habits, exposure to the sun, genetical susceptibility to develop skin cancer and immunosuppression [3] are known risk factors of lip squamous cell carcinoma.

Carcinomas of the lip can be successfully treated with different methods: surgery, external beam radiotherapy (EBRT) and brachytherapy. The choice of treatment depends on the tumor size, the location and the expected functional and esthetic results of each option, but it also depends on treatment type accessibility. There are no randomized studies comparing these different treatment strategies.

Retrospective data have clearly shown the efficacy of low dose rate (LDR) interstitial brachytherapy with Iridium-192 wires as curative treatment in lip cancer with only 5–10% local failures at 5 years and with very satisfying cosmetic results [4,5,6]. Compared to EBRT, brachytherapy has the advantage of delivering a high localized dose with rapid fall-off and short treatment duration, without the need of additional margins taking into account target movements [7]. This high dose and ability to spare critical normal tissues cannot be achieved by any external beam radiotherapy technique. Interstitial implant therapy is ideal for selectively delivering a high dose exclusively to the primary tumor volume, thus minimizing sequelae. Compared to surgery, brachytherapy is particularly appropriate for periorificial tumor locations, such as tumors involving the oral mucosa, the lip or the nose, as long as no extension to the bone is noted [5,8].

Following the switch from Iridium wires to after-loading machines, several authors have published their experience with high dose rate (HDR) brachytherapy in lip carcinomas, showing satisfactory oncological and functional results [5]. Pulsed dose rate (PDR) treatment is another modality combining the physical advantages of HDR technology (optimization and radiation safety) with some radiobiological advantages of conventional continuous LDR brachytherapy in terms of normal tissue sparing [5]. The PDR technique was demonstrated as an effective as well as safe treatment method with excellent functional and cosmetic results in head and neck tumors [9].

The aim of this study was to investigate the complications and local efficacy in patients treated with PDR brachytherapy in our institution.

## 2. Materials and Methods

### 2.1. Patients Characteristics

We retrospectively examined the clinical records of all consecutive patients treated by brachytherapy between October 2010 and December 2019 at our institution (Gustave Roussy, Villejuif, France) for a histologically confirmed invasive carcinoma of the lip. Patients could receive brachytherapy either as primary treatment or as adjuvant in the case of positive margins after initial surgery. Bone invasion was an exclusion criterion for brachytherapy. According to the clinical situation (tumor stage according to the AJCC Cancer staging manual, 8th edition, patient age and comorbidities) and risk for lymph node extent, brachytherapy could be associated to a surgical staging of the neck: surveillance was usually proposed for cutaneous T1 SCC, sentinel lymph node was proposed in squamous T2 SCC and mucosal T1 to T2 tumors, and patients with T3 tumors usually underwent upfront lymph node dissection. 

In the case of radiologically and/or histologically demonstrated lymph node extension, brachytherapy could be combined with lymph node dissection and/or external beam radiotherapy treating the neck. The treatment was decided after multidisciplinary discussion and approved by our head and neck tumor board. This retrospective study was conducted in accordance with ethical standards, and a database was formally declared. Ethical committee approval was obtained (n° 2020-57), and the Helsinki declaration of human rights was observed.

### 2.2. Brachytherapy Procedure

**Figure 1:**
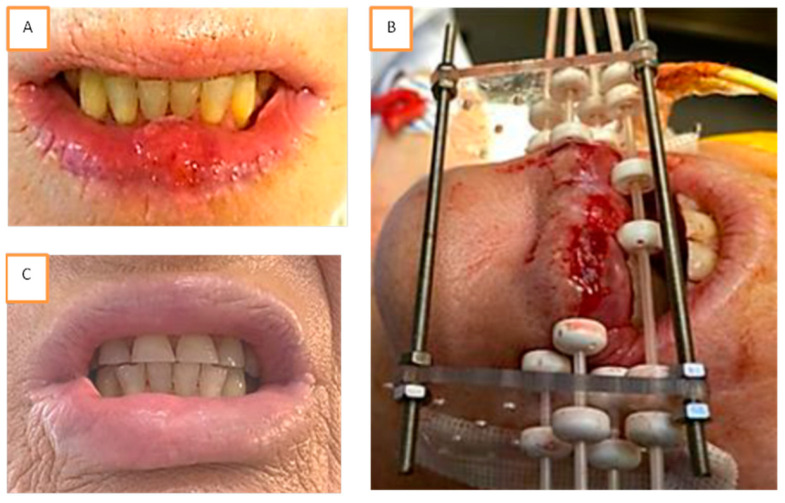
Example of brachytherapy implantation for a T1 N0 squamous cell carcinoma of the inferior lip. Tumor was measured at 18 × 15 mm² and localized in the mid lip (**A**). Implantation was performed following Paris system rules with two planes. Four needles were used, including one needle in plesiotherapy to increase the dose at the level of the tumor (**B**). The patient experienced acute reaction with radiomucitis grade II at week 6. At week 23, complete response was achieved with disappearance of all acute reactions and without any sequalae (**C**).

**Figure 2:**
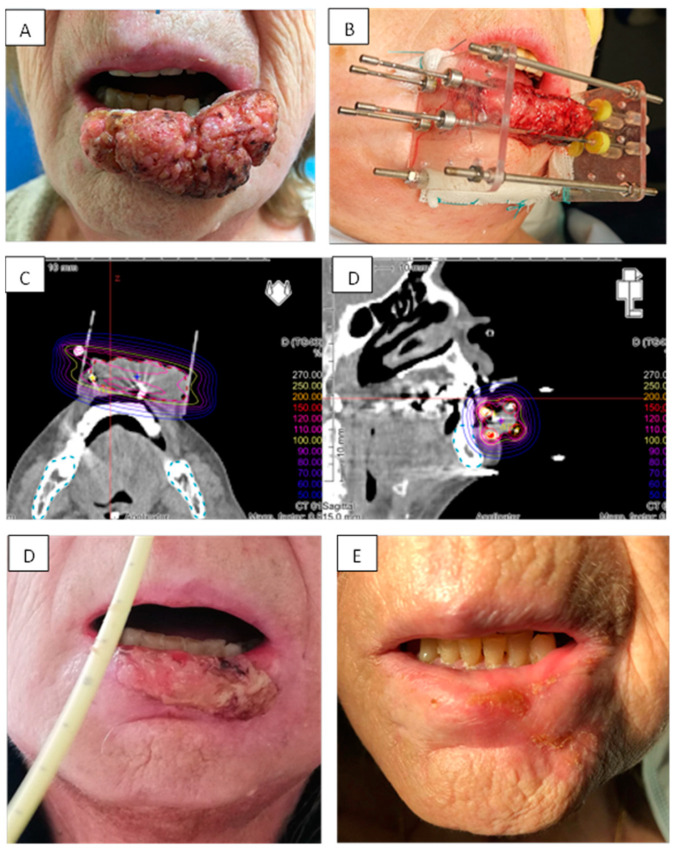
Example of brachytherapy implantation for a T3 N0 squamous cell carcinoma of the inferior lip. Tumor was measured at 60 mm and localized in the inferior lip (**A**). Implantation was done following Paris system rules with two planes arranged in squares with 15 mm spacing. Four needles were used (**B**). The dose distribution was optimal, with high dose to the tumor (>70 Gy) while minimizing dose to the bone (**C**). Patient experienced acute reaction with radiomucitis grade II at week 8 (**D**). At one year, complete response was achieved with disappearance of all acute reactions and persistence of a small fibrotic scare (**E**).

The implantation was always performed following strict asepsis in an operating theatre. During the anesthesia procedure, a nasogastric tube was placed and kept during the hospitalization in order to facilitate feeding. The implant was performed under general anesthesia with horizontally placed plastic catheters or needles in a parallel, quadratic or triangular distribution according to the Paris system rules with distances between the sources ranging from 9 to 18 mm (Figure 1 and Figure 2). The implantation of interstitial needles was performed in order to appropriately cover the macroscopic tumor plus a 5–10 mm safety margin. In non-well limited tumors, the margins could be even more than 10 mm, and the whole lip could be included. If the tumor was exophytic, one to two catheters could be placed with one part in the air outside the tumor surface by means of an outer distance tubing (plesiotherapy). The implant was maintained with an applicator, possibly made of two square plates of Plexiglass, with perforated equidistant holes allowing a perfect parallelism between needles or plastic tubes. Then, patients underwent a 3D Computed Tomography scan. The images were transferred to Plato^®^ or Oncentra^®^ (Nucletron, an Elekta company, Stockholm, Sweden) for treatment planning. The dimensional volume was digitized on the treatment system. Active positions within catheters were chosen in order to appropriately cover the target volume. Dose was prescribed to the reference isodose, which corresponded to 85% of the basal isodose. Treatment was delivered through continuous hourly pulses of 0.42 Gy per pulses, keeping the daily dose to 10 Gy. No dose constraint was used for the mandible, but slight optimization could be performed in order to keep the 100% isodose outside the external mandibular tabula. The minimal dose delivered to the most exposed 0.01cc, 0.1cc and 2cc of the mandible was recorded (respectively, D_0.01cc_, D_0.1cc_, D_2cc_), as well as the Total Reference Air Kerma (TRAK). The PDR brachytherapy was delivered through the PDR Selectron afterloader (Nucletron, Elekta Company, Stockholm, Sweden). For the entire treatment duration, patients were asked to wear a personalized lead dental protection, built after a systematic dental oncology consultation. Removal of the needles was performed under conscious sedation.

### 2.3. Follow-Up

All patients were systematically seen after 6 weeks to monitor acute reactions and early complications. Patients were then followed by clinical examination every 3–4 months for the first two years, every 6 months for years 3 and 4 and by annual visits thereafter. Patients were examined with biopsy or appropriate radiological imaging if there was any suspicion of abnormality. Biopsies were not systematic, as they increase the risk of necrosis. Procedure complications, acute toxicities, and clinically relevant late toxicities (defined as toxicity occurring or lasting more than six months following brachytherapy) were systematically recorded and graded in accordance with Common Terminology Criteria for Adverse Events (CTCAE v4.0). We conducted a retrospective quality of life assessment. For this purpose, the Functional Assessment of Chronic Illness Therapy system of Quality of Life questionnaire for Head and Neck cancer (FACT H&N) version 4 was used in its French version [10]. Patients were interviewed by phone by two radiotherapists trained in brachytherapy.

Univariate analyses were performed by univariate Cox models in R v4.0.2 (The R Core Team 2019, http://www.r-project.org/index.html and accessed on 1 December 2020). Descriptive survival plots using Kaplan and Meier curves were reported.

## 3. Results

### 3.1. Patients Characteristics and Treatment Data

From October 2010 to December 2019, 38 patients were treated in our institution for a lip carcinoma by PDR brachytherapy. The patients’ characteristics are detailed in Table 1. The median age was 73, and the majority of patients were males (*n* = 29). The majority of patients were treated for T1 or T2 tumors (*n* = 30). Twenty-six patients received brachytherapy as primary intent treatment, and twelve patients underwent prior surgery with positive margins. Fifteen patients underwent lymph node exploration, consisting of either sentinel node detection (four patients underwent SLND prior to brachytherapy) or lymph node dissection (five patients prior to brachytherapy, five patients after brachytherapy and one patient prior and after brachytherapy). Four patients with suspicious lymph nodes at diagnosis underwent lymph node dissection, following multimodal assessment and multidisciplinary discussion taking into account the clinical examination and imaging results. At the time of analysis, the mean and median follow-up times were 35.4 and 20.6 months (range: 0.2–101 months), respectively. Two patients were rapidly lost to follow-up (patients refused post-treatment follow-up) and were excluded from the toxicity and survival analysis. The median total dose was 70.14 Gy (range: 60–85 Gy) (Table 2). Two patients received 60 Gy. One of them had undergone surgery two months before brachytherapy for a synchronous vestibular squamous cell carcinoma with the concomitant excision of the lip tumor. The second one received brachytherapy as adjuvant treatment following surgery with positive margins. One patient needed 15 catheters to cover a bifocal lower lip tumor.

### 3.2. Toxicity

Almost all patients underwent radiomucitis grade II or higher after brachytherapy (Table 3). All patients recovered in a maximum of 6 months. Examples of radiomucitis are presented in Figure 1 and Figure 2. At final follow-up, only three patients suffered from fibrosis grade II generating moderate lip retraction, but no patient had impairment in lip continence. None of those three patients had been treated with prior surgery. Grade I fibrosis was encountered in eighteen patients with no lip function impairment. Five of them had received prior local surgery. Fifteen patients presented absolutely no clinical evidence of fibrosis and no symptom related to treatment.

One female patient presented grade 2 chronic pain, localized at the level of the lip and starting three months after brachytherapy. She was treated at the total dose of 69.5 Gy after primary lip surgery. Three patients presented late grade 1 pain with no systematic need for pain relievers, and the pain spontaneously resolved after 18 months. Seventeen patients presented grade I depigmentation with no impact on cosmetic results. No mandible necrosis was reported.

### 3.3. Quality of Life Analysis

Only 11 patients could be reached to answer the FACT H&N questionnaire and responded. They were first asked to quote between 0 (not at all) and 5 (definitively) if they would recommend the brachytherapy treatment. In this subgroup of patients, 9 out of 11 stated they would definitively recommend the brachytherapy treatment. For all FACIT scales and symptom indices, the higher the score, the better the QoL (Table 4). The median FACT H&N total score was 127 out of 148, and the median FACT H&N Trial Outcome Index was 84 for a maximum of 96. The Trial Outcome Index is most likely to be changed by an intervention being tested. Among these 14 patients, 10 out of 11 had presented acute radiomucitis grade II or more that had totally resolved, and 7 out of 11 had late fibrosis grade I or more. Patients who had received prior local surgery to brachytherapy (*n* = 2) presented lower scores with a mean FACT H&N Trial Outcome Index at 61.6 and a mean FACT H&N total score at 91.

### 3.4. Tumor Control Analysis

**Figure 3:**
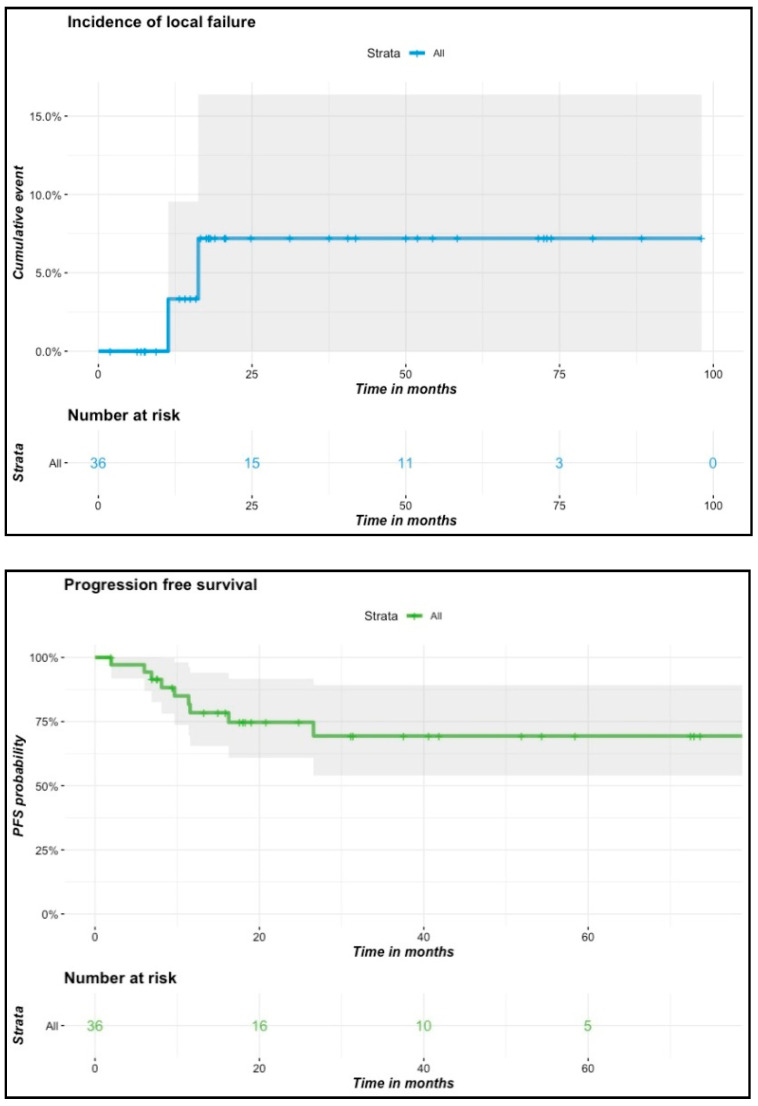

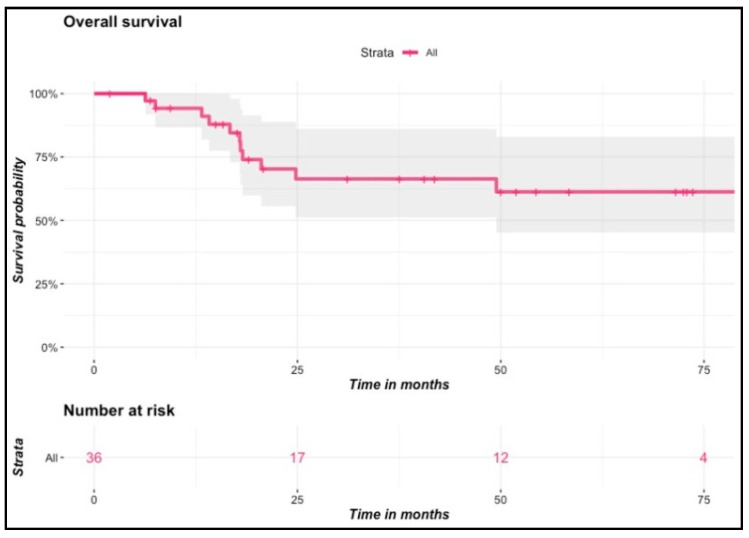
Incidence of local failures, progression-free survival and overall survival curves.

At 6-week follow-up, 35 out of 36 patients presented a complete clinical response. During the follow-up, two patients had local failure, diagnosed 11 and 16 months following brachytherapy, respectively. Both of them presented initially T1N0 tumors and were treated at doses of 65 and 70 Gy, respectively. One of them received prior surgery with positive margins before brachytherapy. The second had a past history of surgery for a cheek tumor. He relapsed on the contralateral part of the lip seven years after primary treatment. Seven patients presented lymph node progression in the neck during the follow-up. Among them, three patients had undergone nodal exploration prior to or after brachytherapy, and two of them underwent additional lymph node external irradiation. At final follow-up, 12 patients died at a median age of 79 years (range: 59–90). Six of them died from the evolution of their lip cancer, and the remaining six patients died from other causes. None of the clinical variables or dosimetric parameters was found to be predictive of local failure, progression-free survival or overall survival in univariate or multivariate Cox regression analyses. Survival and incidence curves of local failure and progression-free survival are presented in Figure 3. The Kaplan–Meier estimated probability of local failure was 7.2% (95% CI: 0.84–1) at two and at four years. Four-year estimated probabilities of progression-free survival and overall survival were 69.4% (95% CI: 0.54–0.89) and 66.4% (95% CI: 0.51–0.86), respectively. Among patients with initially T3 or T4 tumors (*n* = 7), none of them experienced local failure, but one patient experienced tumor progression in the cheek outside the primary tumor bed associated with lymph node progression one year after treatment. Four of those presented lymph node progression after a median follow-up time of 7.5 months (range: 6–26.6 months) that led to disease-specific death for three of them. Two of them had undergone a primary lymph node dissection and none had received EBRT. There was no difference according to tumor site (lower versus upper lip carcinoma).

## 4. Discussion

The choice between the different lip cancer treatments depends on the tumor size, its localization and on the expected functional sequelae of surgical treatment. The treatment goal is not only to cure the cancer but also to preserve function, good mouth opening and closing of the lips, and good cosmetic results. Small lesions (T1 tumors) can be treated by surgery or radiation therapy with the same outcome in terms of survival. However, regarding local control, tumors > 1 cm have a high probability of local relapse after surgery alone, occurring in 10–15% of cases, depending on tumor size [11,12]. Up to 30% of patients with T1 lip SCC treated with upfront excision have close (<5 mm) or positive margins, which is a major factor for local relapse, combined with tumor size, grade and peri-neural invasion [13]. Although most local failures can be salvaged, the treatment of local recurrence (with second surgery or external radiotherapy) is potentially associated with long-term sequelae. Adjuvant radiotherapy improves local control, though associated with significant morbidity and technical issues in terms of target repositioning during fractionated radiotherapy. The mobility of the lip during the session imposes large safety margins, leading to more bone and soft tissue toxicities. A wedge surgical excision can be used in infiltrative tumors, but lip function may be impaired [14,15]. Reconstructive surgery could provide good results, but the periorificial location may be challenging to preserve lip function [16]. Furthermore, most lip tumors occur in elderly patients, with frequent comorbidities and poor general health conditions.

Brachytherapy consists of the placement of radioactive material directly inside the tumor. Compared to any other external radiotherapy technique, brachytherapy provides the best ratio between tumor dose and normal tissue sparing. LDR brachytherapy performed using iridium 192 (^192^Ir) wires showed very good results in terms of local control, functional and cosmetic outcomes in lip cancers [4,5]. In 2014, the production of ^192^Ir wires stopped. Moreover, LDR brachytherapy potentially exposes medical and para-medical staff to radiation protection issues. Some teams decided to treat lip tumors using high dose rate (HDR) brachytherapy with good results. With HDR brachytherapy, irradiation is delivered through a few high dose fractions over a few days. From a biological perspective, HDR is supposed to be inferior to continuous LDR in terms of normal tissue sparing. Very good local control could be achieved with HDR brachytherapy, up to 100% for T1 tumors, while T2 tumors achieved 93.2% local control [17]. In a study including 103 patients [18], Ghadjar et al. found no differences in local recurrence-free survival, regional recurrence-free survival and overall survival rates at five years between patients treated either by LDR or HDR brachytherapy. No difference in terms of acute or late toxicities was found. It should be noted than 61% of the patients were treated for T1 tumors. Another study [19] revealed than patients treated with HDR brachytherapy were more likely to develop radiomucitis grade III compared to LDR (33 vs. 23%). In contrast, Guinot et al. [20] analyzed a cohort of 104 patients who underwent HDR brachytherapy compared to 99 who underwent LDR brachytherapy, and reported equal effectiveness in local control and disease-free survival, but fewer complications arose when using HDR. This may be a consequence of a higher capability of dose optimization with stepping source technology to minimize hyperdose sleeves and bone structure. It should also be noted that in this publication there was a very high unexplained incidence of complications with LDR brachytherapy, with 15% of soft tissue necrosis versus none in our study [20].

PDR brachytherapy combines the physical advantages of HDR in terms of optimization abilities and radiation safety with the theoretical radiobiological advantages of hyperfractionation [5]. Isodose volumes in tissues can be created flexibly by a combination of the careful placement of the catheter and the adjustment of the dwell times of the computerized stepping source [21]. Few studies have been published using PDR brachytherapy in lip cancers. Johansson et al. [22] conducted a retrospective study of 43 patients with primary or recurrent clinical T1-T3N0 lip cancers. The 2-, 5- and 10-year rates of actuarial local control were 97.6, 94.5 and 94.5%; overall survival 88.0, 58.9 and 39.1%; disease-free survival 92.7, 86.4 and 86.4%, respectively. Long-term side effects were mild, and the cosmetic outcome was excellent, except for one case (2%) of soft tissue necrosis and one case (2%) of osteoradionecrosis. In another retrospective study evaluating the clinical outcomes of 32 patients, local control was achieved in 93.5% of the patients, and good or excellent functional/cosmetic results were obtained in 28 (90%). The actuarial local control at 5 years was 94% (100% for T1 tumors), and 5-year all-cause overall survival was 73% [23]. The largest series on PDR use for head and neck tumors was published by Strnad and colleagues, who published their experience on 385 patients with various tumor sites. With a median follow-up of 63 months, 5-year local relapse-free survival was 85.8%, while 5-year overall survival was 68.9%. Serious late side effects, such as soft tissue or bone necrosis, were observed in 10.2 and 4.9% of patients, respectively [24].

In our study, we reported a Kaplan–Meier estimated local failure probability of 7.2% at two and four years. The four-year estimated probabilities of progression-free survival and overall survival were 69.4 and 66.4%, respectively. Half of the deaths were related to other causes. The outcome results are very good and comparable to those from other studies. We observed low late morbidity, as only three patients suffered from grade II fibrosis, and none had lip continence impairment, despite a high rate of acute grade III radiomucitis. All radiomucites resolved within three months following local topic treatment. It should be noted than in our institution, we systematically used a nasogastric tube during the entire hospitalization time, and we assessed the necessity to maintain this at home with nutritional follow-up for the time of acute side effect recovery. These low rates of late toxicities could be partially explained by the use of a personalized lead dental protection and optimization procedure on bone structures, as shown by low doses to the mandible.

To our knowledge, our study is the first focusing on the quality of life assessment after lip brachytherapy. We observed a very high score rate, which was partially lowered by patients undergoing surgery prior to brachytherapy. Patients who had local surgery prior to brachytherapy (*n* = 2) had lower scores with a mean FACT H&N Trial Outcome Index of 61.6 and a mean FACT H&N total score of 91. These findings could be explained by the fact that the preservation of lip function and esthetic aspects is easier if cumulative morbidity of surgery and brachytherapy is avoided, while anatomy is restored at integrum in most cases of exclusive brachytherapy. Esthetic damage recovery was also very good and rapid to provide good patient self-satisfaction. Moreover, our results highlight the fact that large tumors (T3-T4) are also good candidates for PDR lip brachytherapy with excellent local control and few side effects (Figure 2). All seven patients with tumors > T2 achieved complete response and local control. According to the guidelines from the Groupe Européen de Curiethérapie -European Society for Therapeutic Radiology and Oncology for brachytherapy of head and neck carcinomas, updated in 2016 [8], lip tumors < 5 cm can be treated with brachytherapy as sole treatment. A wedge excision may be practiced in very limited tumors < 5 mm, but if negative margins cannot be achieved, brachytherapy could offer better functional and cosmetic results than surgery or external irradiation alone.

Our study is negatively impacted by retrospective biases and a low number of patients, preventing us from examining more thoroughly the impact of treatment parameters and determining predictive factors. First, we should highlight the low number of patients included and that the sample is inhomogeneous. The median follow-up was short, in relation to the fact that numerous patients were referred to our center for brachytherapy, as patients preferred to be followed by their referringradiation oncologist or head and neck surgeon, with difficulties of obtaining updates on a regular basis, especially as the cohort comprised elderly patients: 13 out of 38 (34%) were aged ≥ 80 years at the time of brachytherapy, including four patients aged ≥ 90 years. Furthermore, 11 out of 38 patients (29%) were treated in the final two years, and nine patients died of tumor progression, with a median survival time of 17 months. Therefore, long-term survival and QoL data were not available. Our results would, in theory, deserve multicentric, comparative and prospective evaluations. A prospective assessment during different treatment schedules would be a better approach to monitor the QoL evolution. We also note the fact that, as recommended, all potential candidates for brachytherapy should undergo a detailed examination of the head and neck region before any treatment [25]. Node recurrences represented an important part of loco-regional failure in our study. In a large study including 617 patients, the lymph node involvement ranged from 7.9% in T1-T2 to 27.9% in T3-T4 [26], and in another article with 299 patients, it ranged from 5.6% in T1-T2 to 17.6% in T3 [27]. Although most primary tumors can be treated with brachytherapy alone (provided that there is no bone involvement), elective cervical treatment should be considered in lip tumors larger than 10 mm or with skin or commissural involvement or with a pattern of tumor invasion or in the case of a tumor developing from the labial mucosa [28,29]. However, the management of the clinical cN0 neck in the SCC of the lip is controversial, with some authors suggesting elective neck dissection in almost all cases [11,30], whereas others prefer a wait-and-see management [31]. More recently, a sentinel node biopsy (SNB) procedure was proposed, with a high rate of sentinel lymph node (SLN) localization (range: 90–93%), and rates of micrometastases ranging from 7.1 to 16.6% [32]. The sentinel node technique can make the elective treatment easier and less aggressive than radiation or cervical neck dissection, and it could be performed as a “one day” procedure [33].

## 5. Conclusions

This study on the long-term outcome of PDR brachytherapy as treatment of lip cancer shows an excellent local control rate with very limited late side effects. Long-term QoL analysis and survival data are warranted to compare with historical LDR data. All tumors without bone invasion should be considered as good candidates for primary treatment with brachytherapy. A multimodal approach should help to improve regional control, which remains a major concern in patients with most advanced disease (T3, T4).

## Figures and Tables

**Table 1:** Patient characteristics (*n* = 38).

Characteristics		*n*/Median	Min–Max/%
Age		73	35–92
Gender			
	F	9	23.7
	M	29	76.3
Performance status			
	0	17	44.8
	1	20	52.6
	2	0	0
	3	1	2.6
Tobacco			
	0	17	44.7
	withdrawn	16	42.1
	active	5	13.2
Histology			
	squamous cell carcinoma	37	97
	polymorph adenocarcinoma	1	2.6
Tumor localization			
	lower lip	28	73.7
	upper lip	8	28
	lip and cheek	2	5.3
T			
	1	14	36.9
	2	16	42.1
	3	7	18.4
	4	1	2.6
*n*			
	0	34	89.5
	1	2	5.3
	2	2	5.3
M			
	0	38	100
	1	0	0

**Table 2:** Dosimetric data.

Treatment Data	Median	Min–Max	*n*
Catheters number	4	15–2	38
Plans number	2	4–1	37
Catheters spacing	13	18–9	37
Length activation	65	50–120	38
Dose/pulse (cGy)	42	40–50	38
Pulse number	166.5	120–179	38
Total dose (Gy)	70.14	60–85	38
V 100% (cm^3^)	16.49	4.7–47.8	34
V 150% (cm^3^)	4.45	2.3–14.1	33
V 200% (cm^3^)	2.1	1–5.8	33
TRAK (mGy)	1.82	0.77–3.36	37
mandible D2cc (Gy)	28.6	7.2–49.4	31
mandible D0.1cc (Gy)	42.6	9.1–82.8	31
mandible D0.01cc (Gy)	48.4	11.8–101	31

TRAK: Total Reference Air Kerma; D0.01cc, D0.1cc, D2cc: minimal dose delivered to the most exposed 0.01cc, 0.1cc and 2cc parts of the mandible, respectively; V100, 150, 200: volume receiving 100, 150 and 200% of the prescription isodose, respectively.

**Table 3:** Acute and late toxicity reports (*n* = 36).

Toxicity Data (Grade)		*n*	%
Radiomucitis			
	G0	0	0%
	G1	2	6%
	G2	19	53%
	G3	15	42%
Odynophagia			
	G0	20	56
	G1	13	36
	G2	3	8
	G3	0	0
Fibrosis			
	G0	15	42
	G1	18	50
	G2	3	8
	G3	0	0
Chronic pain			
	G0	32	89
	G1	3	8
	G2	1	3
	G3	0	0
Depigmentation			
	G0	19	53
	G1	17	47
	G2	0	0

**Table 4:** Quality of life assessment (*n* = 11).

Quality of Life Assessment		*n*/Median	%/Min–Max
Would You Recommend the Treatment?			
	0 (not at all)	0	0%
	1	0	0%
	2	0	0%
	3-neutral	0	0%
	4	2	18%
	5 (definitively)	9	82%
FACT H&N scores (range)			
	Physical well-being score (0–28)	25	15–27
	Social/family well-being score (0–28)	26.8	1.2–28
	Emotional well-being score (0–24)	19	14–24
	Functional well-being score (0–28)	23	11–28
	Head and Neck cancer subscale (0–40)	36	25–40
	FACT H&N Trial Outcome Index (0–96)	84	58.2–94
	FACT H&N total score (0–148)	127	75.4–146

FACT H&N: Functional Assessment of Chronic Illness Therapy system of Quality of Life questionnaire for Head and Neck cancer.

## Data Availability

Data are unavailable due to patients confidentiality.
